# Suicide After Contact With Child and Adolescent Mental Health Services—A National Registry Study

**DOI:** 10.3389/fpsyt.2022.886070

**Published:** 2022-05-09

**Authors:** Helene Astrup, Martin Ø. Myhre, Anine T. Kildahl, Fredrik A. Walby

**Affiliations:** National Centre for Suicide Research and Prevention, Institute of Clinical Medicine, University of Oslo, Oslo, Norway

**Keywords:** suicide, mental health services, adolescent, children, mental disorders, registry study, inpatient, outpatient

## Abstract

**Background:**

Research has shown a strong association between suicide and mental disorders, and people in contact with services for mental health and substance use are known to be at high risk of suicide. Still, few studies have previously described suicide among young people in contact with Child and Adolescent Mental Health Services.

**Aim:**

The aim of this study is to examine the prevalence of contact and suicide rates by gender and age groups, and to describe patient demographics and service utilization in secondary mental health services.

**Methods:**

All young people in contact with Child and Adolescent Mental Health Services in the year prior to death in the period 2008–2018 were identified by linking the Norwegian Cause of Death Registry and the Norwegian Patient Registry. We estimated the prevalence of contact and suicide rates among those with and without contact, by gender and age groups. Characteristics of treatment contact were compared between boys and girls. Variables with significant differences were entered into a multivariate logistic regression model using gender as an outcome.

**Results:**

More girls (39.7%) than boys (11.8%) had contact with Child and Adolescent Mental Health Services in the year prior to death. Among girls, suicide rates per 100,000 patients increased linearly in the age groups 10–13, 14–16, and 17–19 years: 5, 22, and 38 per 100,000 patients, respectively. Among boys, the suicide rate increased sharply from 7 per 100,000 patients in the age group 14–16 years to 40 per 100,000 patients in the 17–19-year-old group. In the age-adjusted multivariate model, boys were 4.07 (1.22–14.44, *p* = 0.024) times more likely to have terminated contact at the time of death.

**Conclusion:**

This study shows gender differences in both suicide rates and service utilization among young people in contact with Child and Adolescent Mental Health Services before suicide, and future studies should focus on identifying the causes of these gender differences in service contact.

## Introduction

Suicide is one of the leading causes of death in young people (1, 2). The suicide rate among 10–19-year-olds is estimated to be ~4 per 100,000—with large variations according to sex, age and country of residence (3). Suicide among children younger than 15 years is rare, and the prevalence rises during teenage years and into adulthood, especially among boys (3–5). As in the adult population, suicide rates are higher among boys than girls in most countries (3, 6). In several high-income countries (UK, US, Canada, and Australia), an increase in suicide rates among adolescents has been observed over the past decade (7–9). According to the latest WHO estimates, suicide is the fourth most common cause of death in young people worldwide (10), and suicide among adolescents is a major public health concern that should receive attention in research and be addressed by means of national suicide preventive strategies.

Self-harm refers to intentional self-poisoning or self-injury, irrespective of motive or the extent of suicidal intent (11), and is one of the strongest risk factors for suicide (12, 13). With an estimated overall prevalence of 16.9 %, self-harm is considerably more common among adolescents than suicide (14), and has increased considerably among adolescents during the past decades (14, 15). Given that suicide in children and adolescents is a very rare event, many studies have used self-harm, suicidal behaviors or suicidal ideation as proxy variables to examine suicide, but since risk factors and populations are far from overlapping, it is important to examine suicide in these groups directly.

Suicide is strongly associated with mental disorders (16), and this association has also been found in studies in adolescents, especially among older adolescents (13, 17, 18). However, as with suicide among adults, the issue of suicide among adolescents is complex, and the causes are only partly understood. Genetic and biological factors, social and environmental factors, family-related factors, adverse life events and psychological aspects are important risk factors (1, 11, 19). Among adolescents, psychosocial factors such as family related problems, bereavement, academic pressure, bullying, relationship problems, excessive drinking and illicit drug use, physical health conditions, and suicide-related internet use are found to be important risk factors (5, 13, 18). A lower prevalence of psychosocial risk factors, self-harm, and mental disorders has been found among boys compared to girls prior to suicide (5). Some studies have showed that the youngest children, below 16 years of age, present less apparent warning signs and were less exposed to known risk factors before suicide than older adolescents (17, 20).

Children and adolescents who die by suicide are more often in contact with mental health services compared to controls (21). Some studies have found that the prevalence of contact with services for children and adolescents before suicide is lower than what is found among adults (22, 23). Rodway et al. (13) found that 32 % of all adolescents who died by suicide in England had lifetime contact with child and adolescent mental health services before suicide, with more girls having contact with services before suicide than boys.

Most studies on service contact before suicide in children and adolescents have either used wide age groups or included broader definitions of service use (5, 21, 23–26), and national registry studies on suicide in Child and Adolescent Mental Health Services (CAMHS) are currently lacking (22). The use of complete national samples over a broad time period is important, and the availability of national health registries provides a unique opportunity, especially when studying rare events such as suicide in children and adolescents. Consequently, the aim of the current national registry study is to describe characteristics and service utilization among young people who have been in contact with CAMHS in the year before suicide. First, we aim to examine the prevalence of contact, and suicide rates by gender and different age groups. Second, our aim is to describe patient demographics and service utilization in secondary mental health services during the year prior to death. Our hypothesis is that we will find gender differences in these variables for boys and girls who had contact with CAMHS during the year prior to death.

## Materials and Methods

### Sample and Design

This study used a registry-based cross-sectional observational design. The sample consisted of a linkage between the Norwegian Cause of Death Registry (NCDR) (27) and the Norwegian Patient Registry (NPR) (28). First, a list of all deaths by suicide and deaths by undetermined intent (X60-84; Y10-34; Y87.0; Y87.2) between Jan 1, 2008, and Dec 31, 2018, was obtained from the NCDR. These were then linked to data from mental health services and substance use disorder services in the NPR using a unique 11-digit personal identifier. All young people who had been in contact with CAMHS within the year before their date of death were included in the final sample (*n* = 73). The sample included two cases aged 20 years, and nobody was older than 20 years. The NPR conducted the data linkage.

The aggregated total number of young people in contact with CAMHS in the year prior to death was retrieved from the NPR, and the aggregated total number of suicides in Norway in young people aged between 10 and 19 years was retrieved from the NCDR. Aggregated data was grouped by gender and age groups.

### Data Sources

The NCDR (27) contains, among other things, information about dates of death and causes of death in Norway. The registry has a high coverage, with medical information for >98 % of all deaths (29).

Secondary health services, including private institutions and specialists contracted to the regional health authorities, routinely report all patient activity (including administrative data, patient demographics, and medical information such as treatment and diagnoses) to the NPR (28). Personally identifiable information has been included in the registry since 2008, and data from substance use disorder services has been included since 2009. In Norway, the healthcare system, including secondary mental health services, is publicly funded and is accessible at a low deductible fee through referral. Child and Adolescent Mental Health Services (CAMHS) is a separate secondary mental health service in Norway, serving children and adolescents up to 18 years of age. It is possible to receive treatment in CAMHS until the age of 23, if treatment started before the age of 18. CAMHS mainly offers outpatient treatment, but also includes inpatient services. Service utilization during the last year was retrieved from child and adolescent mental health services, adult mental health services, substance use disorder services, and private mental health practitioners.

## Measurements

### Demographic Information and Diagnosis

Information about gender, age, date of death, and ICD-10 codes for method of suicide were retrieved from the NCDR. Age was used as both a continuous and a categorical variable. Mental health diagnoses according to ICD-10 (30) registered after contact with secondary mental health services and substance use disorder services in the year prior to death were retrieved from the NPR. Diagnoses were collapsed into the categories of substance use disorders (F10-F19), psychotic disorders (F20-F29), affective disorders (F30-F39), anxiety disorders (F40-F48), behavioral syndromes associated with physiological disturbances and physical factors (F50-F59), personality disorders (F60-F69), development disorders (F70-F79, F80-F89), and behavioral and emotional disorders with onset usually occurring in childhood and adolescence (F90-F98). Unspecified diagnoses (Z-diagnoses, R-diagnoses and F99 diagnosis) and no diagnoses were collapsed into one category. No diagnoses of organic mental disorder (F00-F09) were present in the material.

### Service Utilization

Information regarding service utilization was retrieved from the NPR and analyzed based on two time periods. First, we assessed data at the last contact. Level of care was recorded at two levels: outpatient contact and inpatient contact at last contact. Contact status at last contact was recoded into two levels: ongoing contact—contact as outpatient with open referral and contact within 90 days or current inpatient contact (discharged from inpatient services as deceased); and terminated contact—last contact as outpatient with closed referral or no contact within 90 days or discharged from inpatient services as alive. Service utilization in the year prior to death includes the number of those who, in addition to contact with CAMHS, had contact with adult mental health services (including private mental health specialists) and/or substance use disorder services. The number of those admitted to inpatient care (including admissions in CAMHS, adult mental health services, and substance use disorder services) and the number of those with outpatient contact in these services were also included. The total number of outpatient contacts during the year prior to death were counted for each person.

### Analyses

Data was analyzed with R version 4.1.2 (31). To assess the prevalence of contact with CAMHS in the year prior to death, we compared data for the children and adolescents in the age groups 10–13, 14–16, and 17–19 years in the current study with data of all children and adolescents who died by suicide in the same age groups during the study years from the NCDR. Two cases aged above 19 years were excluded from the analysis regarding prevalence of contact and suicide rates (Table 1), but were included in the other analyses regarding characteristics and service utilization (Tables 2, 3). Suicide rates in children and adolescents with contact were calculated by standardizing the number of suicides with the number of children and adolescents who had contact with CAMHS during the year prior to death. Suicide rates in children and adolescents without contact were calculated by standardizing the number of suicides with the number of children and adolescents without contact in CAMHS. Confidence intervals for the rates were estimated using the Poisson distribution, and age was collapsed into three categories-−10–13, 14–16, and 17–19 years—and analyzed separately for boys and girls.

**Table 1:** Number of suicides and suicide rates in children and adolescents with and without contact in Child and Adolescent Mental Health Services (CAMHS) in the year prior to death.

		**Contact with child and adolescent mental** **health services in the year prior to death**			**No contact with child and adolescent mental health services in the year prior to death**		
	**Age**	***n* ^a^**	**%**	**Rate^b^ (95% CI)**	***n***	**%**	**Rate^c^ (95% CI)**
**Girls**							
	10–13	3	37.5	5.04 (−0.66 to 10.74)	5	62.5	0.39 (0.05 to 0.74)
	14–16	19	52.8	22.36 (12.31 to 32.41)	17	47.2	1.82 (0.95 to 2.68)
	17–19	26	33.8	38.08 (23.44 to 52.72)	51	66.2	5.24 (3.80 to 6.68)
	N	48	39.7	22.60 (16.2 to 28.90)	73	60.3	2.29 (1.77 to 2.82)
**Boys**							
	10–13	2	25.0	1.83 (−0.71 to 4.37)	6	75.0	0.46 (0.09 to 0.84)
	14–16	5	13.5	6.70 (0.83 to 12.57)	32	86.5	3.19 (2.08 to 4.29)
	17–19	16	10.7	39.82 (20.31 to 59.33)	134	89.3	12.52 (10.40 to 14.65)
	N	23	11.8	10.30 (6.07 to 14.50)	172	88.2	5.11 (4.25 to 5.87)

a *The total sample includes two cases aged 20 years and they are excluded from the analyses in Table 1*.

b *Rate per 100, 000 patients in CAMHS*.

c *Rate per 100, 000 population without contact in CAMHS*.

**Table 2:** Description of all young people in contact with Child and Adolescent Mental Health Services (CAMHS) in the year prior to death by gender.

	**Boys**	**Girls**	**Total**		
	**(*n =* 24)**	**(*n =* 49)**	**(*n =* 73)**	**OR^a^ (95% CI)**	***P***
Age, mean (sd)	16.7 (1.9)	16.3 (1.7)	16.5 (1.8)	–	0.248
Age groups					0.301
10–13	2 (8.3)	3 (6.1)	5 (6.8)	1.06 (0.13–7.03)	
14–16	5 (20.8)	19 (38.8)	24 (32.9)	0.42 (0.12–1.27)	
17–20	17 (70.8)	27 (55.1)	44 (60.3)	1 (ref)	
**Method of suicide**, ***n*** **(%)**					
Hanging or strangulation	14 (58.3)	32 (65.3)	46 (63.0)	0.75 (0.25–2.31)	0.612
Jumping from a height or jumping/lying in front of a moving object	5 (20.8)	8 (16.3)	13 (17.8)	1.34 (0.30–5.42)	0.747
Other	5 (20.8)	9 (18.4)	14 (19.2)	1.17 (0.27–4.54)	1.00
**Mental disorders during the year prior to death**, ***n*** **(%)**					
Substance use disorders (F10-F19)	1 (4.2)	6 (12.2)	7 (9.6)	0.32 (0.01–2.85)	0.414
Psychotic disorders (F20-F29)	1 (4.2)	3 (6.1)	4 (5.5)	0.67 (0.01–8.89)	1.00
Affective disorders (F30-F39)	10 (41.7)	26 (53.1)	36 (49.3)	0.64 (0.21–1.89)	0.457
Anxiety disorders (F40-F48)	4 (16.7)	18 (36.7)	22 (30.1)	0.35 (0.07–1.28)	0.106
Behavioral syndromes associated with physiological disturbances and physical factors (F50-F59)	2 (8.3)	6 (12.2)	8 (11.0)	0.66 (0.06–4.07)	1.00
Personality disorders (F60-F69)	0	9 (18.4)	9 (12.3)	–	0.026
Development disorders (F70-F79, F80-F89)	0	2 (4.1)	2 (2.7)	–	1.00
Behavioral and emotional disorders with onset usually occurring in childhood and adolescence (F90-F98)	6 (25.0)	11 (22.4)	17 (23.3)	1.15 (0.30–4.07)	1.00
No diagnosis/only unspecified diagnosis	9 (37.5)	9 (18.4)	18 (24.7)	2.63 (0.76–9.17)	0.089
**Service utilization at last contact**, ***n*** **(%)**					
Child and adolescent mental health services	21 (87.5)	39 (79.6)	60 (82.2)	1.78 (0.40–11.17)	0.525
Adult mental health services^b^	3 (12.5)	7 (14.3)	10 (13.7)	0.86 (0.13–4.26)	1.00
Substance use disorder services	0	3 (6.1)	3 (4.1)	–	0.546
**Level of care at last contact**, ***n*** **(%)**					
Outpatient contact	23 (95.8)	42 (85.7)	65 (89.0)	1 (ref)	
Inpatient contact	1 (4.2)	7 (14.3)	8 (11.0)	0.26 (0.01–2.27)	0.258
**Contact status at last contact**, ***n*** **(%)**					
Ongoing contact	12 (50.0)	41 (83.7)	53 (72.6)	1 (ref)	
Terminated contact	12 (50.0)	8 (16.3)	20 (27.4)	4.99 (1.49–17.88)	0.005
Days from last contact to suicide, median (IQR)	72.0 (5-186)	6.0 (1-30)	9.0 (2-82)	–	0.008
**Service utilization during the year prior to death**, ***n*** **(%)**					
Adult mental health services^b^	4 (16.7)	17 (23.3)	21 (28.8)	0.38 (0.08–1.41)	0.169
Substance use disorder services	3 (12.5)	15 (20.5)	18 (24.7)	0.33 (0.05–1.36)	0.148
Inpatient contact	5 (20.8)	24 (49.0)	73 (100.0)	0.28 (0.07–0.94)	0.024
Outpatient contact	24 (100.0)	49 (100.0)	73 (100.0)	–	–
Number of outpatient contacts, median (IQR)	13.5 (4.5–15)	21.0 (11-35)	15 (10-31)	–	0.004

a *Reference group is girls*.

b *Adult mental health services and private mental health specialist*.

**Table 3:** Differences in characteristics and service utilization between girls (reference) and boys.

	**Age-adjusted bivariate model**		**Age-adjusted multivariate model**	
**Factors**	**OR (95% CI)**	***p***	**OR (95% CI)**	***p***
**Terminated contact**				
No	1 (ref)		1 (ref)	
Yes	4.99 (1.66–15.99)	0.005	4.07 (1.22–14.44)	0.024
Number of outpatient contacts during the year prior to death	0.98 (0.96–1.00)	0.197	1.00 (0.98–1.02)	0.756
**Inpatient contact during the year prior to death**				
No	1 (ref)		1 (ref)	
Yes	0.22 (0.06–0.69)	0.013	0.26 (0.06–1.01)	0.062

For the categorical variables, differences between genders were tested using Fisher's exact test, since several variables contained small numbers. We used an odds ratio as a measure of the strength of association for boys compared to girls. As age and number of days since last contact were skewed, we tested the difference between groups with a non-parametric Mann-Whitney test, and reported the median for days since last contact. Variables with significant differences (*p* < 0.05) between boys and girls were then entered into a bivariate and multivariate logistic regression model, adjusted for age groups, using gender as an outcome. None of the boys were diagnosed with a personality disorder, and this variable was therefore not included in the model. Since contact status and days from last contact are interdependent, we only included contact status in the multivariate model. Since there were large differences regarding age groups, we adjusted for age groups in the bivariate and multivariate regression models to account for these differences.

### Ethics and Approvals

Given that informed consent could not be retrieved, access to the data was provided by means of an exemption from patient confidentiality granted by the Norwegian Directorate of Health. The Regional Committees for Medical and Health Research Ethics, South-East Norway, approved the project (reference number: 32494). As some numbers will unavoidably be small when reporting on a low-frequency phenomenon, we decided not to stratify the sample with regard to variables other than gender and age groups and gender and suicide methods/service utilization/diagnosis, in order to avoid potential identification.

## Results

### Prevalence of Contact and Suicide Rates

In total, 316 children and adolescents aged 10–19 years died by suicide in Norway between 2008 and 2018. Of those, we identified 73 young people (24 boys and 49 girls) who died by suicide within a year of contact with Child and Adolescent Mental Health Services (CAMHS) during the same period. Overall, 23 % of those who died by suicide were in contact with CAMHS during the year prior to death. There were large gender and age differences, as shown in Table 1. Among the boys, the overall prevalence in contact was 11.8%, compared to 39.7% of the girls. The highest prevalence of contact was among girls in the age group 14–16 years, and the lowest prevalence of contact was among boys in the age group 17–19 years. Of the total sample, 21 were older than 17 years. When excluding these, the prevalence of contact under the age of 18 was 35%. For both genders, as shown in Table 1, suicide rates in CAMHS were highest in the age group 17–19 years. However, suicide rates differed according to age and gender: in girls, the suicide rate gradually increased with age, but in boys, the suicide rate increased more sharply in the oldest age group, and across all age groups the suicide rate in CAMHS was higher among girls than among boys.

### Description of the Sample

Characteristics of the young people who died by suicide within the year of contact with CAMHS are shown in Table 2. The boys/girls ratio was 0.49. The average age was 16.5 years (range 12–20). Only two were 20 years of age. Hanging or strangulation was by far the most frequently used method of suicide in both genders, followed by jumping from a height or jumping/lying in front of a moving object. In total, these two methods accounted for 80% of all suicide deaths among young people who had been in contact with CAMHS during the year prior to death. Two adolescents died by poisoning, and there were no deaths by use of firearms or sharp objects in this study. Two were registered as death by undetermined intent. Affective disorders formed the largest diagnostic group, followed by anxiety disorders. There were 18 (24.7%) who had not received a diagnosis or had only received an unspecified diagnosis during the year prior to death. Significant gender differences (*p* = 0.026) were only found for personality disorders.

### Service Utilization

Most had their last contact with CAMHS (Table 2), but some had their last contact with adult services. The majority were outpatients (current or terminated) at last contact. Eight had their last contact as inpatients, seven of these were current inpatients at the time of death, and fewer than three were inpatients in CAMHS. Significant gender differences were found for contact status at last contact (*p* = 0.005). The majority of girls were registered with an ongoing contact at the time of death, whilst a terminated contact was much more common among boys. The median time from last contact to suicide was 72 days for boys and 6 days for girls (*p* = 0.008).

Although all cases had at least one contact with outpatient services during the year prior to death, girls had a significantly higher number of outpatient contacts (*p* = 0.004). The median number of outpatient contacts was 13.5 for boys and 21.0 for girls. Fewer boys than girls had at least one inpatient contact (*p* = 0.024) during the year prior to death. Contact in adult mental health services was common (28.8%), and 18 (24.7%) had contact in substance misuse services during the year prior to death.

In the age-adjusted bivariate logistic regression model comparing boys with girls, differences in the numbers of those with terminated contact and those with inpatient contact during the year prior to death remained significant (Table 3). Boys were more likely to have terminated contact at the time of death [OR = 4.99 (95% CI 1.66–15.99)], and they were less likely to have inpatient contact during the year prior to death [OR = 0.22 (95% CI 0.06–0.69)]. When adjusted for all other variables, boys were still more likely to have terminated contact [OR = 4.07 (1.22–14.44)].

## Discussion

In Norway, 23 % of those aged 10–19 years who died by suicide in the period 2008–2018 had contact with CAMHS in the year prior to death, with the prevalence of contact being considerably higher among girls. Suicide rates were lowest in the youngest age groups for both genders, and these increased with age, but with a different pattern for boys and girls. In the final multivariate model, boys were four times more likely to have a terminated contact at the time of death.

The contact prevalence in CAMHS is lower than the contact prevalence in adult mental health services and substance use disorder services in Norway, where the proportion of people in contact was well above 40% in all age groups combined (32, 33). Because of the transition into adult mental health services at the age of 18, contact prevalence with CAMHS after the age of 17 is lower, thus affecting the total proportion in contact with CAMHS in the year prior to death. Although the proportion in contact would be 35% if the cases aged 18 or older were excluded from the sample, this is still lower than the proportion in contact with adult mental health services in Norway.

More girls than boys died by suicide after contact with CAMHS. This may be a result of more girls than boys being in contact with CAMHS in the age groups 14–16 and 17–19 years. The suicide rate across all age groups was highest among girls in contact with CAMHS. This is opposite to the trend for the general population (4), the adolescent population (3), and those in adult mental health services (34). Possible explanations for this could be a combination of the lower occurrence of mental disorders among boys in the adolescent population in general (35) and there being less psychosocial risk factors. This could also be a result of a lower prevalence of disclosure of both mental disorders and risk factors among boys, and a lower prevalence of warning signs before suicide, expressed by self-harm, for example (26). It could also be a result of impulsive behavior, and other factors such as help-seeking behavior and the organization of services (potentially more tailored to girls) could also be involved. Moreover, parents in Norway are responsible for providing consent when receiving an offer of treatment for a child under the age of 16. As a result of this, there is a possibility that adolescents at the age of 16 or older may decline an offer of treatment. It is important for the services to reach boys in this age group, as the overall rates are highest in this group.

The suicide rates varied by gender and age groups. Among both those with and those without contact, the suicide rates increased with increasing age. The increase in suicide rates with increasing age may relate to a higher occurrence of mental disorders among older compared to younger adolescents in general (35), and also as shown in studies among adolescents who die by suicide (13, 17, 18). Due to the lower prevalence of mental disorders among children and adolescents who die by suicide (13, 16, 20), together with a high complexity of other psychosocial factors (5, 13, 18) and an overall low proportion in contact with CAMHS in the year prior to death, universal or public health interventions might prove to be the most important approach in order to reduce the number of suicides among adolescents in Norway. Around 70% of all boys and more than 40% of all girls that died by suicide were in the group aged 17–19 without contact in CAMHS, which may illustrate the importance of universal prevention programs for this age group, such as suicide behavior education for young adults (36).

Affective disorders were the most common mental disorder in this study. This is consistent with the literature, where an affective disorder is a known risk factor for suicide in children and adolescents (18), and also a common disorder in the adolescent population (37). More surprising was that few adolescents were diagnosed with a substance use disorder, although a quarter of the sample had been in contact with substance use disorder services during the year prior to death. Thus, under-diagnosis of substance use disorders seems likely, and better assessment and treatment of substance use disorders should be a priority for the services. Furthermore, a high proportion did not receive any diagnosis or had only received an unspecified diagnosis during the year prior to death. Although non-significant, boys were almost three times more likely to receive an unspecified diagnosis compared to girls. Adequate assessment and diagnostics are important prerequisites for the quality of effective treatment, and the high proportion with no diagnosis or an unspecified diagnosis is a cause for concern and makes this an area with potential for improvement. Reservations concerning the diagnosis of children and adolescents could be one explanation for the high proportion without a specific diagnosis. In addition, adolescents may have an unclear pattern of symptoms and comorbidity, and other psychosocial factors may represent a major part of the clinical picture, making it difficult to determine a diagnosis. It is possible that both unassessed personality traits (38) and affective temperament types (39) could also be important.

The finding that everyone in the sample had contact with outpatient services during the year prior to death was anticipated, as outpatient treatment constitutes 95% of the services in CAMHS in Norway (40). The main service utilization factor examined here was that boys who died by suicide were more likely to have terminated contact compared to girls. This finding may explain other significant findings, such as the number of days since last contact and the number of outpatient contacts. Less severe psychopathology or at least fewer warning signs among boys, and perhaps a lower prevalence of help-seeking behavior, and a lower level of engagement in treatment may lead to a premature termination of contact, with potential under-treatment of their problems as a consequence. CAMHS need to be aware of this and individualize the services based on the patient's needs and preferences.

There were no gender differences regarding the method of suicide. Hanging or strangulation was the most frequently used method of suicide, followed by jumping from a height or jumping/lying in front of a moving object. Glenn et al. (3) found the same pattern when examining cross-national suicide rates by suicide method in the same age group. Limited access to certain methods, and suicide contagion (41, 42), in combination with impulsivity, may also have an impact on the choice of method.

In the present study, few children and adolescents died by poisoning or cutting—methods frequently used in non-fatal self-harm (12, 43). This finding is in accordance with earlier studies on adolescents and young people (12, 43), but nevertheless represents important information for clinicians, who regularly assess and treat adolescents with NSSI (non-suicidal self-injury). This also shows the importance of studying suicide in this group directly, and not only through proxy variables.

## Strengths and Limitations

A strength of this study is the use of registry data, which makes it possible to include a complete national sample of all young people who died by suicide within the year after contact with CAMHS. Since the registry data has already been collected for administrative purposes and not by a researcher, it also reduces the risk of ascertainment and recall bias. A major limitation was the low statistical power, which prevents us from examining patient demographics and service utilization considering both gender and age groups simultaneously. Furthermore, some of the results need to be interpreted with caution due to the small numbers and wide confidence intervals. Another major limitation of the current study is the uncontrolled observational design. Consequently, we were unable to estimate the risk associated with the described factors. Furthermore, the registers lack information about previous suicidal behavior, and sociodemographic or social factors. Given our design, we were unable to study the transition into adult mental health services.

## Conclusion

This national registry-based study found that more girls than boys died by suicide during or up to 1 year after contact with CAMHS, and boys were 4 times more likely to have a terminated contact at the time of death. Therefore, it is important for CAMHS to review their services and assess whether they are sufficiently adapted for boys. It is also important to ensure the presence of suicide prevention programs and initiatives that are aimed at engaging boys. Given the large heterogeneity of suicide rates within CAMHS patients, both future studies and efforts at clinical prevention should take into consideration both gender and age groups when it comes to suicide prevention. Nevertheless, the highest suicide risk is among the oldest adolescents, which should be the main target group for suicide prevention in CAMHS. Although both the proportion of those in contact with the services and the suicide rates were much lower than in adult populations, the rates are still much higher among those adolescents who are in contact with CAMHS than those who are not.

Whilst the generalizability of these findings needs to be examined in other countries and health systems, the results could indicate that treatment engagement and retention in CAMHS might be particularly important for boys. For both genders, missing diagnoses of substance use disorders and the frequent use of unspecified diagnoses indicate a potential for quality improvement, which could possibly result in better patient safety. Suicide among young people in contact with CAMHS is an important cause of years of life lost, and future studies should further examine gender differences in treatment utilization and other paths that could guide suicide prevention in children and adolescents.

## Author's Note

Information from the Norwegian Patient Registry has been used in this publication. The authors have sole responsibility for the interpretation and reporting of this data and no endorsement by the Norwegian Patient Registry is intended nor should be inferred. Information from the Norwegian Cause of Death Registry has been used in this publication. The authors have sole responsibility for the analysis and interpretation of this data.

## Data Availability Statement

Data is not publicly available as the individuals are indirectly identifiable. Requests for supplementary data can be made to f.a.walby@medisin.uio.no.

## Ethics Statement

The studies involving human participants were reviewed and approved by the Regional Committee for Medical and Health Research Ethics, South-East Norway (32494). Exemption from patient confidentiality was granted by the Norwegian Directorate of Health. Written informed consent from the participants' legal guardian/next of kin was not required to participate in this study in accordance with the national legislation and the institutional requirements.

## Author Contributions

FW, MM, HA, and AK formulated the research questions, designed the study, and contributed and agreed to the final manuscript. MM curated data. HA analyzed the data and wrote the first draft. FW supervised the project. All authors contributed to the article and approved the submitted version.

## Conflict of Interest

The authors declare that the research was conducted in the absence of any commercial or financial relationships that could be construed as a potential conflict of interest.

## Publisher's Note

All claims expressed in this article are solely those of the authors and do not necessarily represent those of their affiliated organizations, or those of the publisher, the editors and the reviewers. Any product that may be evaluated in this article, or claim that may be made by its manufacturer, is not guaranteed or endorsed by the publisher.

## Abbreviations

CAMHS: child and adolescent mental health services
NCDR: norwegian cause of death registry
NPR: norwegian patient registry
NSSI: non-suicidal self-injury.
